# A self-monitoring wellbeing screening methodology for keyworkers, ‘My Personal Wellbeing’, using an integrative wellbeing model

**DOI:** 10.1186/s12913-023-09213-0

**Published:** 2023-03-14

**Authors:** Garry Elvin, Zeyneb Kurt, Angela Kennedy, Petia Sice, Lee Walton, Paras Patel

**Affiliations:** 1 Department of Computer and Information Sciences, University of Northumbria, Ellison Building, Newcastle Upon Tyne, NE1 8ST UK; 2 Department of Computer and Information Sciences, University of Northumbria, Newcastle Upon Tyne, UK; 3 Cumbria, Northumberland, Tyne and Wear NHS Trust, Newcastle-upon-Tyne, UK

**Keywords:** Staff health, Wellbeing, Integrative wellbeing model, Self-monitoring, Reflective diary, Healthcare

## Abstract

**Background:**

The detrimental impact of Covid-19 has led to an urgent need to support the wellbeing of UK National Health Service and care workers. This research develops an online diary to support the wellbeing of staff in public healthcare in real-time, allowing the exploration of population wellbeing and pro-active responses to issues identified.

**Methods:**

The diary was co-produced by NHS and care stakeholders and university researchers. It was based on an integrative model monitoring mental health symptoms as well as wellbeing indicators. Diary users were encouraged to reflect on their experience confidentially, empowering them to monitor their wellbeing. The data collected was analysed using Mann-Whitney-Wilcoxon and Kruskal-Wallis statistical tests to determine any significant wellbeing trends and issues.

**Results:**

A statistically significant decline in wellbeing (*P* < 2.2E-16), and a significant increase in symptoms (*P* = 1.2E-14) was observed. For example, indicators of post-traumatic stress, including, flashbacks, dissociation, and bodily symptoms (Kruskal-Wallis *P* = 0.00081, 0.0083, and 0.027, respectively) became significantly worse and users reported issues with sleeping (51%), levels of alertness (46%), and burnout (41%).

**Conclusions:**

The wellbeing diary indicated the value of providing ways to distinguish trends and wellbeing problems, thus, informing how staff wellbeing services can determine and respond to need with timely interventions. The results particularly emphasised the pressing need for interventions that help staff with burnout, self-compassion, and intrusive memories.

**Supplementary Information:**

The online version contains supplementary material available at 10.1186/s12913-023-09213-0.

## Background

The recent pandemic of a respiratory virus has posed a significant challenge to the mental health of nations, [1]. Part of the resilience of the nation is based on the capacity of its essential services to continue to function, particularly healthcare. In the UK, there was a concern that the strain of the pandemic may have a detrimental impact on staff wellbeing and both system and personal ‘resilience’. As part of a compassionate response to health workers and key workers, systems needed to be set up to support them. The scope of this new support task was to address any specific psychological disorders that were an understandable response to the challenges. Traditional models of mental health services would screen for disorder and have data on this held by health support services. However, wellbeing is a broader concept of mental health that considers strengths and functioning as well as symptoms. A support service for staff based around wellbeing could work on prevention, address systemic leadership and organisational aspects of mental health, deal with the complexity of the struggles of our colleagues and address ‘disorders’. Self-monitoring was thought to be one way of contributing to such a model. It can aid mindful awareness and empowered choices about when to seek help. Such a model fits better with the concept of trauma informed care that seeks to understand mental health ‘problems’ as survival strategies to a particular context and sees mental health as related to empowerment, linked to personally meaningful goals and based on compassionate, mutual relationships, [2]. This diary emerged from that context, where a regional NHS staff wellbeing hub aspired to develop a service for NHS and social care staff based on empowering and non-stigmatising principles from that available in mainstream mental health services or occupational health teams. That required a novel way of screening staff with focus on self-monitoring of wellbeing rather than mental health diagnosis and a need was identified to develop a diary tool, “My Personal Wellbeing’, to provide people with an anonymous means for monitoring their wellbeing in real time. The diary data could also contribute to an agile service able to respond to staff issues in a proactive way. The tool aims to: determine which factors affect wellbeing; identify the aspects of wellbeing that are improving / declining; identify the aspects of wellbeing that correlate together and may indicate more serious health situations. The diary integrates both mental health symptoms tracking as well as wellbeing indicators monitoring, while similar interventions categorised in the literature typically monitor either symptoms or wellbeing indicators, not both [3–5]. The choice of combining mental health symptoms and wellbeing indicators monitoring was intentional, in order to allow for exploring correlations between changes in symptoms and wellbeing indicators.

The pandemic has had a significant impact on staff. A meta-analysis on the impact on staff mental health during pandemics (n = 38 studies) reported that staff with face-to-face contact with affected patients had greater levels of both acute and post traumatic and stress and psychological distress when compared to lower risk controls, [6]. Similarly, frontline workers in the UK during covid had higher prevalence rates of depression, anxiety and PTSD compared to the rest of the population. In the US, the Panchal et al., [7], survey compared all essential workers in any role or setting compared to non-essential workers, finding essential workers to report higher symptoms of depression and anxiety (42% vs. 30%), the onset or increase of substance use (25% vs. 11%), or to have seriously considered suicide in the past 30 days (22% vs. 8%). In a UK poll of healthcare staff (n = 996) 50% of staff reported that their mental health was impacted because of the Covid-19 crisis, [8].

## Methods

An integrative model of wellbeing was adopted for this study [9], with tracking of symptoms, which refer to the impact of distress on functioning, [10], as well as providing an appreciation of strengths and wellbeing indicators, [11, 12]. Importantly, for healthcare staff in a pandemic, this new tool needed to also incorporate items of work-related impact e.g., burnout, compassion fatigue and vicarious post-traumatic stress disorder [13]. We know that other factors relate to personal resilience too, e.g., meaning to life, [14, 15], sense of threat, [16], self-compassion, [17], sense of connectedness to others, [18], addictions, [19], and moral injury, [20].

The core of the wellbeing model as previously used in assessing the relationship between wellbeing and leadership capability, in the development of a protocol for interoceptive self-awareness in email communication, [21], as well as in evaluating the effect of music listening on wellbeing, [22]. Developing ones awareness of experiences as they unfold includes: witnessing present moment, sensations, bodily states (alert, quiet, pleasant, unpleasant), mental activity (thoughts, feelings, memory, intentions, beliefs, attitudes, etc.) and relational experience (connectedness to others, to nature, etc.), sense of meaning and purpose, and compassionate attitude, [18], ensuring observation nurtures wellbeing as it is conducted in a kind and gentle way, [23]. This has important implications for understanding and evaluation and measurement of human experience.

Thus, a diary method was considered appropriate for this study, [24]. It empowered the participants to monitor and reflect on their own experience while being understanding towards oneself (self-kind), thus supporting recovery, [2, 25].

The impetus for the development of the diary tool emerged from an NHS region’s systems level leadership looking to support the wellbeing of critical NHS and care staff. The diary was co-produced in a process involving NHS staff and university researchers, [26], refined, and trialled for inclusion in the diary. All items were worded with both positive and negative polarity. This enabled both pathology, e.g., severe anxiety, and strengths, e.g., self-compassion, to be tracked. Diary users were encouraged to reflect on their experience and sense of wellbeing, considering the factors identified.

### Participants

The participants were NHS and social care professionals (for example, administrators, care support workers, doctors, nurses, social workers, and student doctors). One hundred individuals participated (Supplementary Table 1). Participants were invited to complete an online diary with 25 questions (Supplementary Table 2) between 14 January and 14 March 2021. In total, 142 diary entries were made, 59 in January, 34 in February, and 49 in March (Supplementary Table 1). Participation was voluntary but it was suggested that users completed the diary once a week.

### Data analysis methods

A non-parametric test, Mann-Whitney-Wilcoxon (Wilcoxon rank sum) was used to run pairwise comparisons for the questions in the diary as there were no prior assumptions regarding the likely average wellbeing scores or the distribution of the data. The pairwise comparisons were made for the diary entries for each month of the trial. In addition, a multiple group comparison was performed to test whether there were any significant wellbeing changes during each of the three months of the trial using a non-parametric test Kruskal-Wallis. The analyses were conducted in R, boxplots were created to illustrate changes in wellbeing using the function *ggboxplot* from the R library *ggplot*.

Correlations between each pair of questions were calculated with the Spearman coefficient using the *cor.test* function in R for each month of the trail. In addition, the aggregated correlations for each question pair were calculated across all three months. Heatmaps were produced using the function heatmap.2 from the R library *gplots* (version 3.1.1).

## Results

Data from one hundred NHS and social care professionals were included in the study. Active participation in the study varied over time with some users completing the diary more regularly than others. This led to an unequal number of records per month during the study period.

### Quantitative analysis of the diary data

**Fig. 1:**
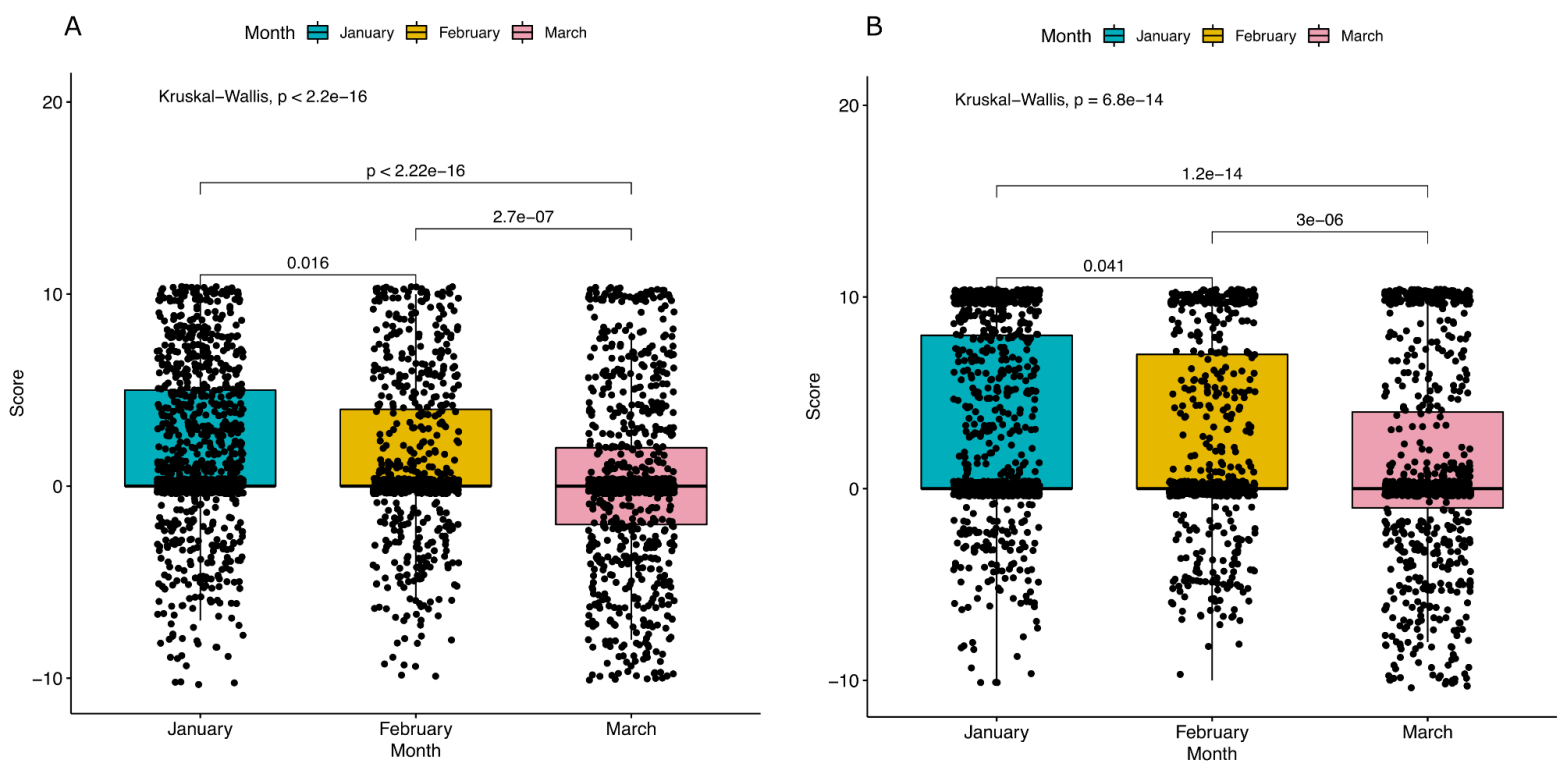
Change in participant scores for (A) “Wellbeing question group” (B) the “Symptom’s question group”

In line with research showing that wellbeing and mental health symptoms are separate concepts, [27], the diary questions were split into two corresponding groups. The questions on, wellbeing included, for example, ones on physical, emotion and cognitive/mental wellbeing. Whereas the questions on symptoms, included, for example, ones on burnout, self-harm and anxiety or worry (Supplementary Table 2 shows the full set of questions). These two groups were analysed separately to examine how mental health and wellbeing in general changed over time. Due to the relatively small sample sizes available for the individual weeks, changes from month to month rather than week to week were analysed. The results can be seen in Fig. 1A and B.

A statistically significant decline in wellbeing was observed (*P* = 0.016 from January to February, *P* = 2.7E-07 from February to March, *P* < 2.2E-16 from January to March). Similarly, there was a significant increase in symptoms over time (*P* = 0.041 for January to February, *P* = 3E-06 for February to March, *P* = 1.2E-14 for January to March).

We found that a significant number of the parameters monitored by the diary showed a decrease in wellbeing and an increase in symptoms (with the spread of data points becoming more negative month by month).

### Individual analysis of the quantitative questions

We investigated each question individually. An initial exploration of the data showed no change for several the questions as users had left the sliders (used to measure scores) at or very close to the mid-point (the default position). In our analysis we only considered the items that exhibited change.

#### Individual wellbeing group question analysis

**Fig. 2:**
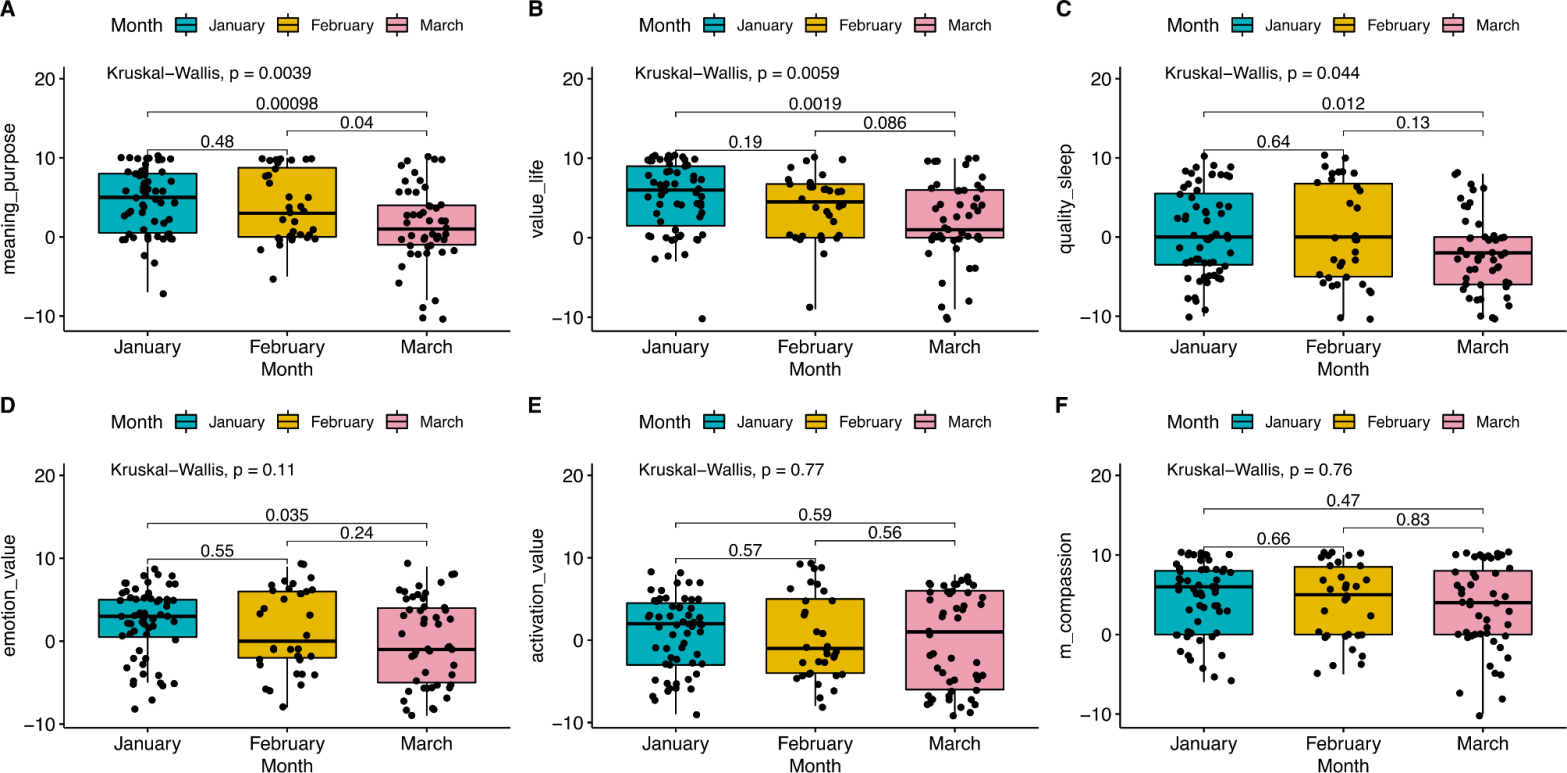
Wellbeing questions with a change in scores. (Statistically significant declines in wellbeing were observed for the ability to complete the activities of daily living, the meaning and value of life, and the quality of sleep (Kruskal-Wallis *P* = 0.0039, 0.0059, and 0.044, respectively for the three months of the trial). The decline regarding daily living was particularly significant from February to March (*P* = 0.04) and for January to March (*P* = 0.00098))

Six of the thirteen wellbeing questions exhibited no change. The questions with a change in scores and those without are shown in Supplementary Table 3A. Figure 2A-F illustrates the changes for these questions - where A represents the *ability to complete the necessary activities of daily living* (m = 3), B *how much meaning and value does life have* (m = 4), C quality of sleep (m=-0.5), D *rating of emotion* (m = 2), E *level of alertness* (m = 1), F *ability to feel empathy or compassion* (m = 5), and where **m**: median score across all participants and months.

Supplementary Table 4 shows that many people experienced wellbeing problems. In particular, people reported issues with sleeping (51%), levels of alertness (46%) and negative emotions (44%).

Overall, the results show a clear decline in wellbeing over the trial, with significant proportions of people reporting problems.

#### Individual symptom group question analysis

**Fig. 3:**
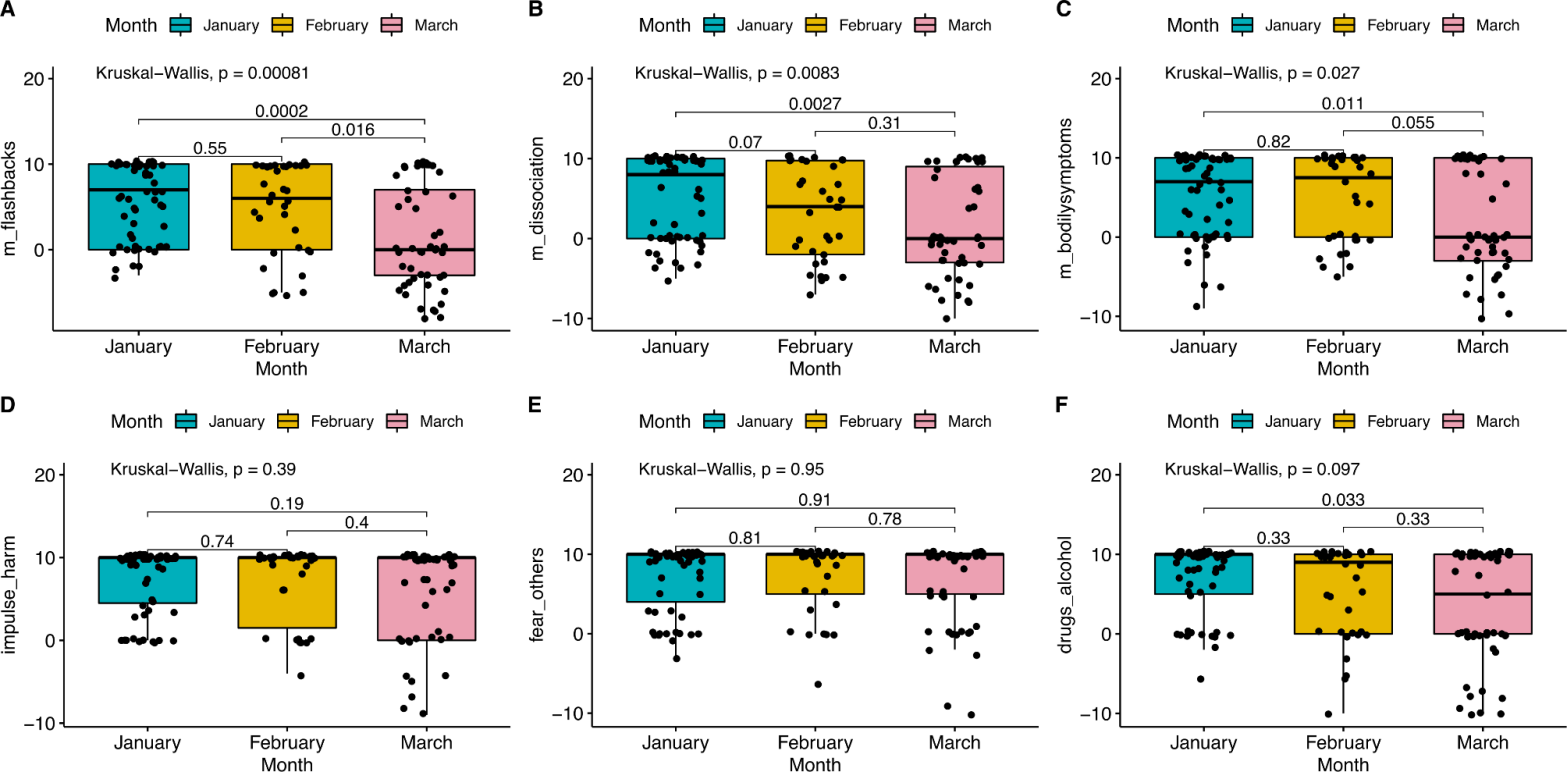
Symptom group questions with a change in scores

Six out of the twelve symptom group questions exhibited no change. The questions with a change in scores and those without are shown in Supplementary Table 3B. Figure 3A-F illustrates the changes for these questions - where A represents *flashbacks* (m = 5), B *dissociation* (m = 4), C *bodily symptoms* (m = 5), D self-harm (m = 10), E risk of harm *from others* (m = 10), F *dependence on drugs or alcohol* (m = 9), and where **m** is the median score across all participants and all time points.

The results suggest that indicators of post-traumatic stress disorder (PTSD), including, flashbacks, dissociation, and bodily symptoms (Kruskal-Wallis *P* = 0.00081, 0.0083, and 0.027, respectively) became significantly worse during the trial. Supplementary Table 5 shows that substantial numbers of people reported problems, particularly flashbacks (22%), feelings of dissociation (32%) and bodily symptoms (23%). In addition, the percentage of people reporting burnout was 41%, anxiety 46%, and struggling to feel pleasure or motivation (a sign of depression), 37%.

In summary, we observed a statistically significant increase in symptoms, including significant numbers reporting burnout, anxiety, and signs of depression.

### The questions with the highest and lowest levels of mental health and general wellbeing

Supplementary Tables 6 and 7 show the ten questions where participants had the highest and lowest levels (respectively) of mental health and general wellbeing during the trial. Relatively few people reported risk of harm from others, self-harm and drug and alcohol dependency. Whereas the poorest levels of wellbeing were for quality of sleep, anxiety/worry and overall emotional state.

The data also suggested improvements in dependency on drugs or alcohol, in empathy / compassion, alertness and physical state, but deteriorations in experience of flashbacks, how fearful people are, and overall emotion scores.

### Impact of different activities on wellbeing and symptoms

**Fig. 4:**
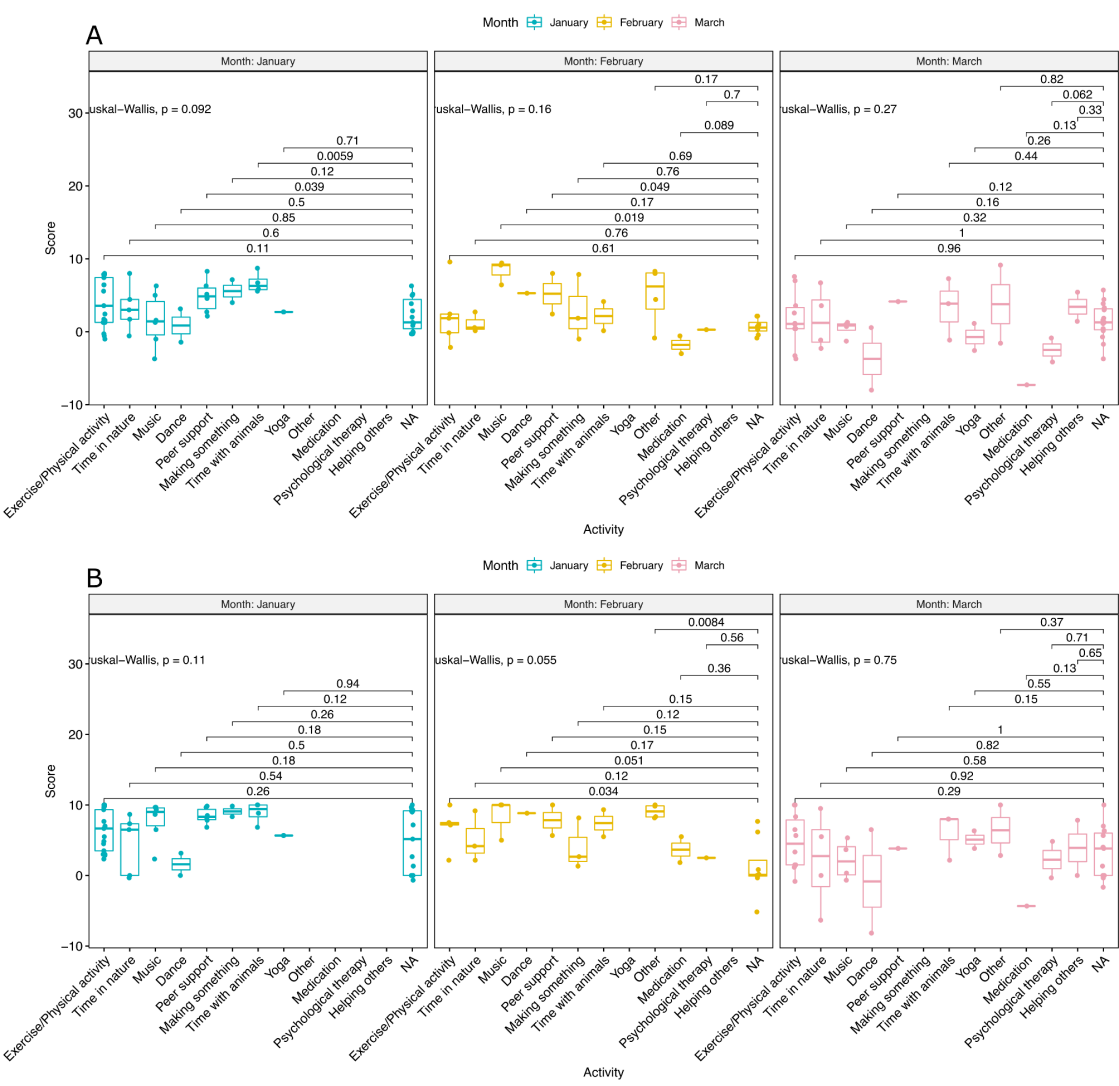
Change in participant scores undertaking activities for (A) *Wellbeing group questions*, (B) *Symptoms group questions*

The impact of different activities, such as exercise (see Supplementary Table 8 for a full list of activities) on wellbeing and symptoms was investigated. The changes in scores were plotted per month (see Fig. 4A and B).

A multi-group comparison (Kruskal-Wallis P > 0.05) showed that overall, the activities did not have a statistically significant impact. *Exercise/physical activity* (*P* = 0.03) and *other* (non-listed) activities (*P* = 0.0084) in February were the only ones to show significant changes (improvements). However, there was obvious trend towards improvement for some of the other activities. For instance, spending *time with animals* (*P* = 0.12, 0.15, 0.15 for January, February, March, respectively), *exercise/physical activity* (*P* = 0.26, 0.03, 0.29 for January, February, March, respectively), and interest in *music* (*P* = 0.18, 0.05, 0.58 for January, February, March, respectively) - especially for those spending *time with animals*. Users who *dance* had statistically significantly higher scores for only February.

The effect of the activities on the *wellbeing* and the *symptoms questions* overall were generally similar. However, where users engaged in *peer support*, they had statistically significantly higher scores for wellbeing (*P* = 0.03, 0.04, 0.12 for January, February, and March).

These results suggest that certain activities during lockdown had the potential to enhance participant wellbeing. They include spending time with animals, musical activity, engaging in peer support, and physical activity.

## Discussion

This method of monitoring wellbeing was developed in response to the need to support health and care staff during the Covid-19 pandemic. The data showed that wellbeing did deteriorate over the winter months of 2021 when the pandemic was in another wave and lockdown was experienced across the UK. This is in line with other research, [28–30]. Factors that were particularly impacted were numerous. People described feeling less able to conduct tasks of daily living. Such functioning is an important aspect of wellbeing in recovery focused models of mental health. Sleep was poor amongst participants and this deteriorated. Poor sleep is problematic because it leaves people physically tired and emotionally volatile, [31]. One participant described it as their “canary in the mine”, meaning they see it as an early warning sign for their own mental health. Meaning to life also deteriorated significantly, suggesting that the barren social landscape of persistent lockdown had impacted on peoples’ sense of value. Interestingly, there is no evidence that suicide has actually increased in this group, [32], but some ideation that life did not have much meaning or value was relatively common.

Professional quality of life is determined by factors such as burnout, vicarious PTSD, and compassion fatigue, [33]. Whilst the wellbeing diary is not diagnostic, a significant number of staff felt tired and numbed from their work. This factor associated with burnout was reported by 41% of people. However, it did not appear to deteriorate. Signs of post-traumatic stress were also very prevalent with this increasing over the three-month period from 10 to 39%. Staff reported an increase in flashbacks showing that such re-experiencing of troubling traumatic events were common, and increases in self-harm, dissociation and experiencing unusual things / having concerning ideas. Compassion fatigue was less prevalent and did not worsen, which shows the values-based resilience of staff in the face of their work-related symptoms. The notable presence of work-related psychological distress is in line with other research, [30], and requires staff support services to address these work-related harms by arranging healing opportunities. PTSD, in particular, can benefit from trauma specific psychological therapies aimed at processing the traumatic events driving the symptoms. Some staff may need to be facilitated to change posts or careers if they feel unable to put themselves in harm’s way repeatedly.

It is interesting that few of the activities that may have been thought likely to have a positive impact on mental health, actually did. Exercise was the activity most associated with positive mental health, although music and animals also helped. It is important to bear in mind that such ‘interventions’ need to form part of the healthy wellbeing culture of our lives even though they can’t be manualised or subject to randomised control trials very easily and items such as dancing would not be as effective on their own in the house rather than with others. Of note is that the main factor to moderate wellbeing was access to support from peers. Peer support models have been importantly rolled out across many services but sometimes such support forms part of the implicit informal relational texture of teams and this is not to be underestimated. Team functioning, time for informal connection and the maintenance of established working alliances is critical to wellbeing [20].

The wellbeing diary was intended as a tool to empower individuals to enhance their awareness of wellbeing. The participants were people working in health, and diary use was voluntary. Participation diminished over time and we speculate that this may have been due in part to staff working under significant pressure during the pandemic and as a result were not easily able to devote time to using the diary. This resulted in a limitation to the study as the number of diary entries per month were unequal. Another factor may have been that the diary was not integrated into the working processes of the organisations.

In a systematic review focused on the implementation and effects of psychological wellbeing interventions in the workplace, Daniels et al. found that learning support structures like mentoring and coaching, and inclusive governance structures were critical to the success of workplace health and wellbeing practices [3].

As a result of the diary, staff wellbeing support services were able to respond with interventions based on needs that emerged, e.g., an insomnia group. The diary shows promise as an alternative way of empowering staff to reflect on their wellbeing and is an potentially valuable resource to show trends in different groups and over time at a population level.

## Conclusion

The wellbeing dairy highlighted the level of distress among participating health and care staff and the need for timely intervention to support their wellbeing. It showed the range, depth, and idiosyncrasy of the interplay between wellbeing factors. Some of the group chose actively and of their own volition to self-monitor themselves. This opportunity to channel motivation for self-awareness over time has not been part of occupational health culture. It represents a shift towards empowerment and a move away from a diagnostic view of mental health. It was very apparent that wellbeing was adaptive and multi-layered. It will be interesting to explore with users of the diary which factors are most relevant to them at various times. It will also be interesting to explore the ongoing impact of the various mitigations. The data showed that factors which did not have a recommendation by NICE (National Institute for Health and Care Excellence) seemed to make the biggest difference, for example, exercise and peer support. Perhaps within a more clinically unwell population of staff this may not hold up so well but generally, these were proving important preventative factors.

The level of engagement with the diary varied by individual over time. This unequal number of diary entries per individual was a limitation on the analysis. However, the diary can help distinguish trends in wellbeing over time and the numbers of staff reporting difficulties in particular wellbeing domains, enabling staff wellbeing services to respond and address these, e.g., putting in place support for addictions (for example, drug and alcohol). It can help ensure that accurate interventions are targeted to at risk staff. This will require services to continue to be agile, innovative about their offers and flexible about meeting staff need with differing individual profiles.

The results particularly emphasised the pressing need for interventions that help staff with burnout, self-compassion, and flashbacks. It also demonstrates the value of population-based wellbeing data that is driven by a trauma informed model of mental health in informing how services can determine and respond to need. Finally, the large proportion of staff with varying areas of distress was obvious. Outreach and engagement will be a key part of any service set up to serve the health and care workforce.

## Electronic supplementary material

Below is the link to the electronic supplementary material.

Supplementary Material 1

## Data Availability

The datasets used and/or analysed during the current study are available from the corresponding author on reasonable request.

## Abbreviations

PTSD: Post Traumatic Stress Disorder
NICE: National Institute for Health and Care Excellence
NHS: National Health Service

## References

[1] Shah K, Kamrai D, Mekala H, Mann B, Desai K, Patel RS. Focus on mental health during the coronavirus (COVID-19) pandemic: applying learnings from the past outbreaks. Cureus. 2020;12(3). 10.7759/cureus.7405 10.7759/cureus.7405 PMC7182052 32337131

[2] Thirkle SA, Kennedy A, Sice PA, Case for (2018). TIC: a Complex Adaptive Systems Enquiry for Trauma Informed Care. Int J Syst Soc (IJSS).

[3] Daniels K, Watson D, Nayani R, Tregaskis O, Hogg M, Etuknwa A, Semkina A (2021). Implementing practices focused on workplace health and psychological wellbeing: a systematic review. Soc Sci Med.

[4] Richardson KM, Rothstein HR. Effects of occupational stress management intervention programs: a meta-analysis. J Occup Health Psychol. 2008;13(1):69. 10.1037/1076-8998.13.1.69 18211170

[5] Lamontagne AD, Keegel T, Louie AM, Ostry A, Landsbergis PA. A systematic review of the job-stress intervention evaluation literature, 1990–2005. Int J Occup Med Environ Health. 2007;13(3):268–80. 10.1179/oeh.2007.13.3.268 17915541

[6] Kisely S, Warren N, McMahon L, Dalais C, Henry I, Siskind D. Occurrence, prevention, and management of the psychological effects of emerging virus outbreaks on healthcare workers: rapid review and meta-analysis. BMJ. 2020;369. 10.1136/bmj.m1642 10.1136/bmj.m1642 PMC7199468 32371466

[7] Panchal N, Kamal R, Orgera K, Cox C, Garfield R, Hamel L, Chidambaram P. The implications of COVID-19 for mental health and substance use. Kaiser family foundation; 2020.

[8] Thomas C, Quilter-Pinner H. Care fit for carers: ensuring the safety and welfare of NHS and social care workers during and after Covid-19. Institute for Public Policy Research; 2020.

[9] Stewart-Brown S, Janmohamed K. Warwick-Edinburgh mental well-being scale. User guide. Version 1; 2008.

[10] Ryan RM, Deci EL (2001). On happiness and human potentials: a review of research on hedonic and eudaimonic well-being. Ann Rev Psychol.

[11] Carr A. Positive psychology: the science of happiness and human strengths. Routledge; 2013.

[12] Seligman ME. Authentic happiness: using the new positive psychology to realize your potential for lasting fulfillment. Simon and Schuster; 2004.

[13] Rommer D (2020). The psychological ill-health of frontline medical staff working with COVID-19 patients: burnout, anxiety, and post-traumatic stress disorder. Psychosociological Issues in Human Resource Management.

[14] Calhoun LG, Tedeschi RG. Posttraumatic growth in clinical practice. Routledge; 2012. 10.4324/9780203629048

[15] Bertolote JM, Fleischmann A, De Leo D, Wasserman D (2003). Suicide and mental disorders: do we know enough?. Br J psychiatry.

[16] Naldi A, Vallelonga F, Di Liberto A, Cavallo R, Agnesone M, Gonella M, Sauta MD, Lochner P, Tondo G, Bragazzi NL, Botto R, Leombruni P (2021). COVID-19 pandemic-related anxiety, distress and burnout: prevalence and associated factors in healthcare workers of North-West Italy. BJPsych Open Cambridge University -Press.

[17] Gilbert P. Mindful compassion: Using the power of mindfulness and compassion to transform our lives. Hachette UK. 2013

[18] Gilbert P (2014). The origins and nature of compassion focused therapy. Br J Clin Psychol.

[19] Chiappini S, Guirguis A, John A, Corkery JM, Schifano F (2020). COVID-19: the hidden impact on mental health and drug addiction. Front Psychiatry.

[20] Guy C, Kunonga E, Kennedy A, Patel P. Moral injury and wellbeing in essential workers during the Covid-19 pandemic: Local survey findings. BMJ. 2022. 10.1136/leader-2021-000518 36170482

[21] Ogwu S, Sice P, Keogh S, Goodlet C. An exploratory study of the application of mindsight in email communication. Heliyon. 2020;6(7);e04305. 10.1016/j.heliyon.2020.e04305 10.1016/j.heliyon.2020.e04305 PMC7364031 32695897

[22] Sice P, Elvin G, Riachy C, Shang Y, Ogwu S, Zink C (2020). Online screening of X-System Music Playlists using an integrative wellbeing model informed by the theory of Autopoiesis. IEEE Access.

[23] Varela FJ, Thompson E, Rosch E. The embodied mind, revised edition: cognitive science and human experience. MIT press; 2017.

[24] Bakker D, Rickard N (2018). Engagement in mobile phone app for self-monitoring of emotional wellbeing predicts changes in mental health: MoodPrism. J Affect Disord.

[25] Sweeney A, Taggart D. (Mis)understanding trauma-informed approaches in mental health. 2018. 10.1080/09638237.2018.1520973 10.1080/09638237.2018.1520973 30345848

[26] Pilemalm S. Participatory design in emerging civic engagement initiatives in the new public sector: Applying PD concepts in resource-scarce organizations. ACM Trans Comput Hum Interact. 2018;25(1):1–26. 10.1145/3152420

[27] Carr A (2019). Positive psychology and you: a self-development guide. Routledge.

[28] Zacher H, Rudolph CW. Individual differences and changes in subjective wellbeing during the early stages of the COVID-19 pandemic. Am Psychol. 2021;76(1):50. 10.1037/amp0000702 10.1037/amp0000702 32700938

[29] Best J. Undermined and undervalued: how the pandemic exacerbated moral injury and burnout in the NHS. BMJ. 2021;374. 10.1136/bmj.n1858 10.1136/bmj.n1858 34326061

[30] Newman KL, Jeve Y, Majumder P. Experiences and emotional strain of NHS frontline workers during the peak of the COVID19 pandemic. Int J Soc Psychiatry. 2021;00207640211006153. 10.1177/00207640211006153 10.1177/00207640211006153 PMC9014765 33845624

[31] Barnes CM, Watson NF. Why healthy sleep is good for business. Sleep Med Rev. 2019;47:112–118. 10.1016/j.smrv.2019.07.005 10.1016/j.smrv.2019.07.005 31450119

[32] Office for National Statistics. *Quarterly suicide death registrations in England* Available: https://www.ons.gov.uk/peoplepopulationandcommunity/birthsdeathsandmarriages/deaths/bulletins/quarterlysuicidedeathregistrationsinengland/2001to2020registrationsandquarter1jantomartoquarter3julytosept. Last accessed 10th December 2021.

[33] Stamm BH. The proQOL manual. Available: https://proqol.org/proqol-manual. Accessed 16th *July* 2021, p.2007.

