# Supplementary material for: A self-monitoring wellbeing screening methodology for keyworkers, ‘My Personal Wellbeing’, using an integrative wellbeing model
Source: BMC Health Serv Res. 2023 Mar 14;23:250. doi: 10.1186/s12913-023-09213-0 (PMC10012319; doi:10.1186/s12913-023-09213-0)
Supplement: Supplementary file 1 — Supplementary Material 1 [file 12913_2023_9213_MOESM1_ESM.docx]

# Supplemental Material

Supplementary Table 1. Total number of entries, participants, and questions

| **Number of total entries in Jan** | **Number of total entries in Feb** | **Number of total entries in Mar** | **Total number of entries** |
| --- | --- | --- | --- |
| 59 | 34 | 49 | 142 |
| **Number of unique participants in Jan** | **Number of unique participants in Feb** | **Number of unique participants in Mar** | **Total number of unique participants** |
| 40 | 27 | 33 | 100 |
| **Number of questions with numeric score** | **Number of questions with free-text fields** | **Other features (demographics, etc)** | **Total number of questions** |
| 37 | 16 | 12 | 65 |

Supplementary Table 2. The questions within the ‘My Personal Wellbeing’ diary.

| **No** | **Variable Name (used in figures)** | **Question** | **Scale** | |
| --- | --- | --- | --- | --- |
|  | | | **Left (-10)** | **Right (+10)** |
| 1 | emotional | Emotional Wellbeing | Poor | Excellent |
| 2 | physical | Physical Wellbeing | Poor | Excellent |
| 3 | Mental | Cognitive/Mental Wellbeing relating to concentration and decision making | Poor | Excellent |
| 4 | relational | Sense of connectedness with others | Lonely, unappreciated | Connected, appreciated |
| 5 | meaning_purpose | Ability to function to achieve necessary activities of daily life | Struggle in most areas | No problems |
| 6 | critical_kind | How self-compassionate are you being towards yourself? | Critical, Shaming, Frustrated | Mindful, Kind, Appreciative, Humanising |
| 7 | fearful_safe | How fearful are you about things currently? | Fearful, Avoidant | Confident, Safe |
| 9 | troubled_actions | Are you troubled by situations that go against good practice or your values? | Troubled | At ease |
| 9 | value_life | How much meaning and value does life have to you? | My life is meaningless, and I have no hope for the future | I feel hopeful and happy to be alive |
| 10 | impulse_harm | Have you done things to hurt yourself to manage feelings? (e.g., cutting) | I cause pain or injury to myself frequently | I don't hurt myself physically |
| 11 | fear_others | Do you think you are at risk of harm from others? | I fear for my life | Safe |
| 12 | quality_sleep | How would you rate the quality of your sleep? | My sleep is disturbed and as a result I am tired | I get enough sleep and feel well rested |
| 13 | drugs_alcohol | Do you depend on drugs or alcohol to get through the day? (Illegal or legal) | Yes, most days | No not at all |
| 14 | compassion | Ability to feel empathy or compassion | Feel burdened by the emotional suffering of others | Able to show and deliver care to others with ease |
| 15 | burnout | Work satisfaction | Feel exhausted, unproductive, or useless at work and would avoid it if I could | Feel energised and rewarded by work |
| 16 | perception | Perception - Experiencing unusual things through my senses or have new concerning ideas | Debilitating | Not at all |
| 17 | flashbacks | Flashbacks - A vivid experience in which you relive some aspects of a traumatic event or feel as if it is happening right now | Debilitating | Not at all |
| 18 | avoidance | Avoidance – Staying away from situations, people, or memories | Debilitating | Not at all |
| 19 | dissociation | Dissociation – Disconnection, numbness, emptiness, or strangely unreal sensations | Debilitating | Not at all |
| 20 | bodily_symptoms | Bodily Symptoms – Sensations or pains in the body that have no medical cause | Debilitating | Not at all |
| 21 | intrusive_thoughts | Intrusive Thoughts – unpleasant ideas in the mind that pre-occupy you | Debilitating | Not at all |
| 22 | anxious_worried | I feel anxious and worried | A lot and it's out of control | At peace |
| 23 | no_interest | I feel so low that I struggle to feel pleasure or motivation | No pleasure in anything | Interested in doing things |
| 24 | emotion_value | Quadrant: Positive -> Negative | Negative | Positive |
| 25 | activation_value | Quadrant: Alert -> Quiet | Quiet | Alert |

Supplementary Table 3. Questions with and without changes in scores for (A) the wellbeing group and (B) the symptom group.

A

| **Wellbeing group questions exhibiting no change** | |
| --- | --- |
| **Label in the figures** | **Question** |
| physical | Physical wellbeing |
| emotional | Emotional wellbeing |
| mental | Cognitive/mental wellbeing - concentration and decision making |
| relational | Sense of connectedness with others |
| critical_kind | How self-compassionate are you being towards yourself? |
| fearful_safe | How fearful are you about things currently? |
| **Wellbeing group questions exhibiting a change** | |
| **Label in the figures** | **Question** |
| meaning_purpose | Ability to function to achieve necessary activities of daily living |
| value_life | How much meaning and value does life have to you? |
| quality_sleep | How would you rate the quality of your sleep? |
| Compassion | Ability to feel empathy or compassion |
| emotion_value | Negative to positive |
| activation_value | Quiet to alert |

B

| **Symptom group questions exhibiting no change** | |
| --- | --- |
| **Label in the figures** | **Question** |
| Burnout | Work satisfaction |
| Avoidance | Staying away from situations, people, or memories |
| Intrusivethoughts | Unpleasant ideas in the mind that pre-occupy you |
| anxious_worried | I feel anxious and worried |
| no_interest | I feel so low that I struggle to feel pleasure or motivation |
| troubled_actions | Are you troubled by situations that go against good practice or your values? |
| **Symptom group questions exhibiting change** | |
| **Label in the figures** | **Question** |
| flashbacks | A vivid experience in which you relive some aspects of a traumatic event or feel as if it is happening right now |
| Dissociation | Disconnection, numbness, emptiness, or strangely unreal sensations |
| Bodilysymptoms | Sensations or pains in the body that have no medical cause |
| impulse_harm | Have you done things to hurt yourself to manage feelings? |
| fear_others | Do you think you are at risk of harm from others? |
| drugs_alcohol | Do you depend on drugs or alcohol to get through the day? |

Supplementary Table 4. Percentage of people reporting problems via the wellbeing questions.

| **Question** | **Percentage of people reporting problems** | | | |
| --- | --- | --- | --- | --- |
|  | January | February | March | All months |
| Ability to function to achieve necessary activities of daily life | 8% | 9% | 33% | 17% |
| How much meaning and value does life have to you? | 10% | 6% | 19% | 12% |
| Quality of sleep | 55% | 41% | 56% | 51% |
| Level of emotional wellbeing | 35% | 44% | 53% | 44% |
| Level of alertness | 45% | 47% | 47% | 46% |
| Ability to feel empathy or compassion | 18% | 13% | 17% | 16% |

Supplementary Table 5. Percentage of people reporting problem reporting symptoms.

| **Question** | **Percentage of people reporting symptoms** | | | |
| --- | --- | --- | --- | --- |
|  | January | February | March | All months |
| Flashbacks | 10% | 19% | 39% | 22% |
| Dissociation - disconnection, numbness, emptiness, unreal sensations | 23% | 34% | 42% | 32% |
| Bodily symptoms - sensations or pains that have no medical cause | 15% | 19% | 36% | 23% |
| Have you done things to hurt yourself to manage feelings? | 0% | 3% | 17% | 6% |
| Do you think you are at risk of harm from others? | 5% | 3% | 11% | 6% |
| Drugs/alcohol dependency | 5% | 13% | 25% | 14% |

Supplementary Table 6. The 10 questions with the highest number of people reporting positive responses.

| **Question** | **Entries** | | **Rank** | | |
| --- | --- | --- | --- | --- | --- |
|  | Feb | Mar | Feb | Mar | Change |
| Do you think you are at risk of harm from others? | 65 | 118 | 1 | 1 | None |
| Have you done things to hurt yourself to manage feelings? | 64 | 113 | 2 | 2 | None |
| Do you depend on drugs or alcohol to get through the day? | 57 | 99 | 6 | 3 | +3 |
| Perception - experiencing unusual things / concerning ideas | 62 | 98 | 3 | 5 | -2 |
| How much meaning and value does life have to you? | 58 | 98 | 4.5 | 5 | -0.5 |
| Ability to feel empathy or compassion | 55 | 98 | 7.5 | 5 | +2.5 |
| Ability to function to achieve the activities of daily life | 55 | 90 | 7.5 | 7 | +0.5 |
| Experiencing flashbacks | 58 | 85 | 4.5 | 8 | -3.5 |
| Bodily symptoms – sensations / pains with no medical cause | 51 | 80 | 9 | 9 | None |
| Dissociation - disconnection, numbness, emptiness, unreal sensations | 49 | 77 | 10 | 10 | None |

Supplementary Table 7. The 10 questions with the lowest number of people reporting positive responses.

| **Question** | **Entries** | | **Rank** | | |
| --- | --- | --- | --- | --- | --- |
|  | Feb | Mar | Feb | Mar | Change |
| How would you rate the quality of your sleep? | 37 | 74 | 1 | 1 | None |
| I feel anxious and worried | 35 | 72 | 2 | 2 | None |
| Emotional wellbeing from poor to excellent | 34 | 70 | 3 | 3 | None |
| How self-compassionate are you being towards yourself? | 32 | 64 | 4 | 4 | None |
| Burnout - level of work satisfaction | 29 | 63 | 5.5 | 5 | +0.5 |
| Avoidance - staying away from situations, people, or memories | 28 | 61 | 7 | 6 | +1 |
| Physical wellbeing | 29 | 59 | 5.5 | 7.5 | -2 |
| How fearful are you about things currently? | 25 | 59 | 9.5 | 7.5 | 2 |
| I feel so low that I struggle to feel pleasure or motivation | 25 | 53 | 9.5 | 9 | +0.5 |
| Intrusive thoughts – unpleasant ideas in the mind that pre-occupy you | 20 | 52 | 13 | 10.5 | +2.5 |

Supplementary Table 8. List of the activities performed for wellbeing.

| Exercise/Physical activity | Peer support | Music | Making something |
| --- | --- | --- | --- |
| Time in nature | Medication | Dance | Helping others |
| Time with animals | Psychological therapy | Yoga | Other |

Supplementary Table 9. Participants and questions with a score change that is strongly correlated by time.

| Question | # of the participants with a high correlation (\|r\| >0.4) | List of participant IDs (separated by ';') | Corr score per person (separated by ';') |
| --- | --- | --- | --- |
| Emotional | 5 | 11; 36; 54; 161; 165 | -0.702; 1; 0.949; 0.866; 0.866 |
| troubled_actions | 5 | 11; 36; 54; 161; 165 | -0.42; -0.866; 0.7; 0.5; 0.866 |
| impulse_harm | 5 | 11; 36; 35; 161; 165 | -0.588; 0.866; 0.82; -0.866; 0.866 |
| m_compassion | 5 | 11; 36; 54; 161; 165 | -0.636; -1; 0.821; -0.866; -0.866 |
| m_bodilysymptoms | 5 | 11; 36; 35; 161; 165 | -0.564; -1; 0.845; -0.866; 0.866 |
| m_intrusivethoughts | 5 | 11; 36; 35; 161; 165 | -0.458; 0.866; -0.441; 0.866; 0.866 |
| no_interest | 5 | 36; 54; 35; 161; 165 | -0.5; 0.447; -0.455; 0.866; 0.866 |
| emotion_value | 5 | 36; 54; 35; 161; 165 | 0.866; 0.447; 0.638; 0.866; 0.866 |
| Physical | 4 | 11; 36; 161; 165 | -0.566; 0.866; 0.5; 0.866 |
| Mental | 4 | 36; 54; 161; 165 | 0.5; 0.894; 1; -0.866 |
| meaning_purpose | 4 | 11; 36; 161; 165 | -0.582; 1; 0.5; -0.866 |
| critical_kind | 4 | 36; 54; 161; 165 | 0.866; 0.791; 0.5; -0.866 |
| value_life | 4 | 11; 36; 161; 165 | -0.62; 0.866; 0.866; 0.866 |
| burnout | 4 | 11; 54; 161; 165 | -0.492; 0.738; 0.866; -0.866 |
| m_perception | 4 | 11; 35; 161; 165 | -0.562; 0.778; 0.866; -0.866 |
| m_avoidance | 4 | 36; 54; 161; 165 | 0.5; 0.707; 0.866; -0.866 |
| Relational | 3 | 36; 161; 165 | 1; 1; 0.866 |
| fearfull_safe | 3 | 54; 161; 165 | 0.975; 0.5; -0.866 |
| fear_others | 3 | 11; 35; 161 | -0.53; 0.441; -1 |
| quality_sleep | 3 | 36; 35; 161 | -1; 0.577; 0.866 |
| drugs_alcohol | 3 | 11; 54; 161 | -0.673; 0.527; -0.866 |
| m_flashbacks | 3 | 11; 35; 165 | -0.562; -0.638; -0.866 |
| m_dissociation | 3 | 36; 161; 165 | 0.5; 0.866; 0.866 |
| anxious_worried | 3 | 36; 35; 161 | -0.5; 0.464; 0.866 |
| activation_value | 3 | 36; 54; 165 | -1; 0.975; -0.866 |
| Motivated | 2 | 11; 54 | -0.598; 0.707 |
| Stimulated | 2 | 11; 54 | -0.462; 0.707 |
| Frustrated | 1 | 161 | -0.866 |
| Anxious | 1 | 161 | -0.866 |
| Enthusiastic | 1 | 11 | -0.5 |
| Content | 1 | 36 | 0.866 |

**FIGURES**


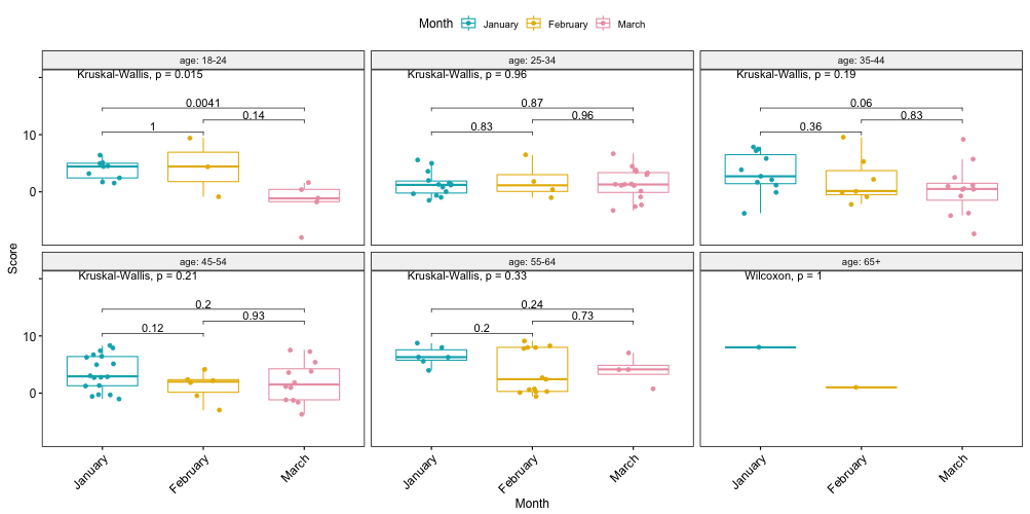


Supplementary Figure 1. Change in scores for the Wellbeing questions by “age group”.


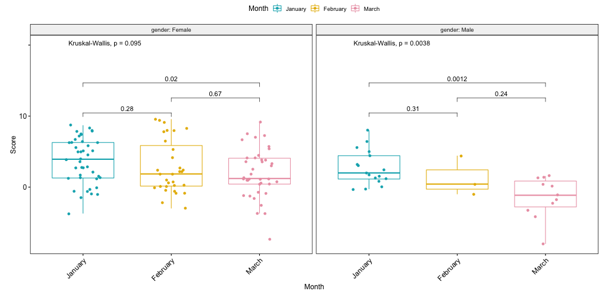


Supplementary Figure 2. Change in scores for the Wellbeing questions by “gender”.


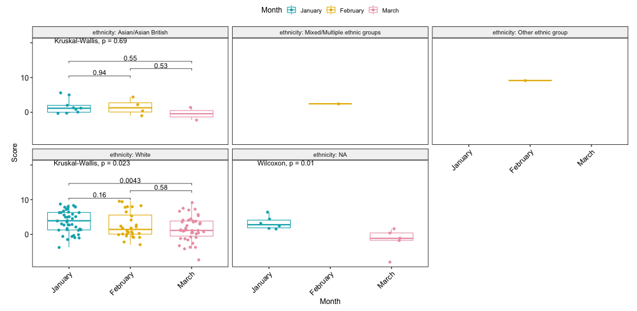


Supplementary Figure 3. Change in scores for the Wellbeing questions by “ethnicity”.


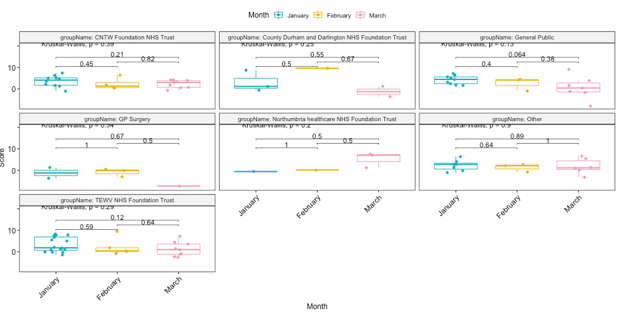


Supplementary Figure 4. Change in scores for the Wellbeing questions by “NHS group”.


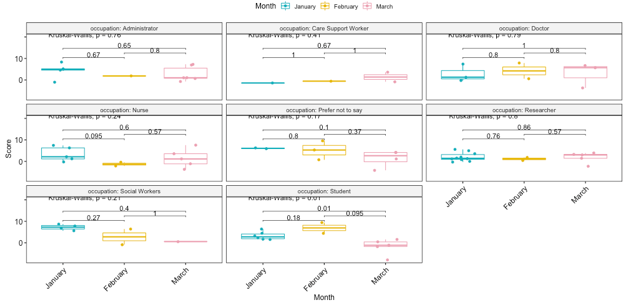


Supplementary Figure 5. Change in scores for the Wellbeing questions by “occupation type”.


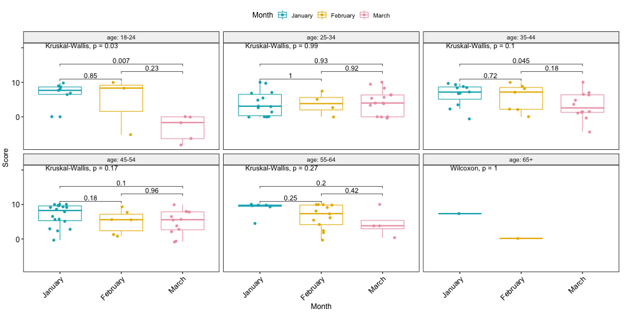


Supplementary Figure 6. Change in scores for the Symptoms questions by “age group”.


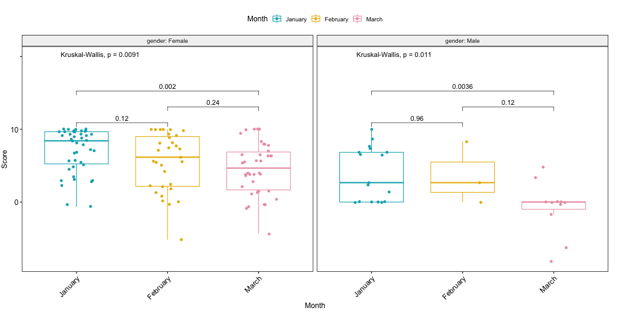


Supplementary Figure 7. Change in scores for the Symptoms questions by “gender”.


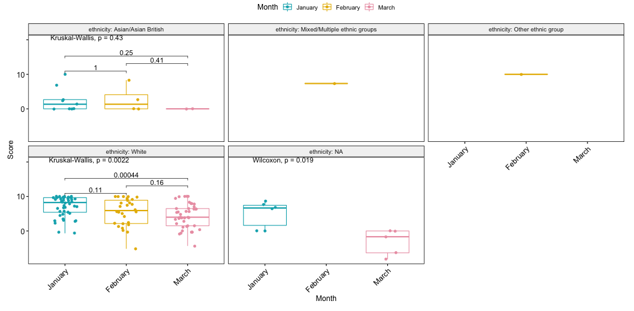


Supplementary Figure 8. Change in scores for the Symptoms questions by “ethnicity”.


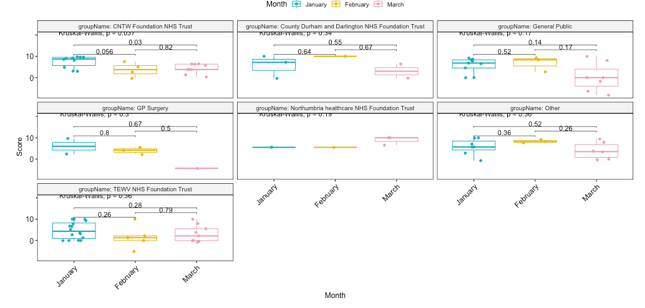


Supplementary Figure 9. Change in scores for the Symptoms questions by “NHS group”.


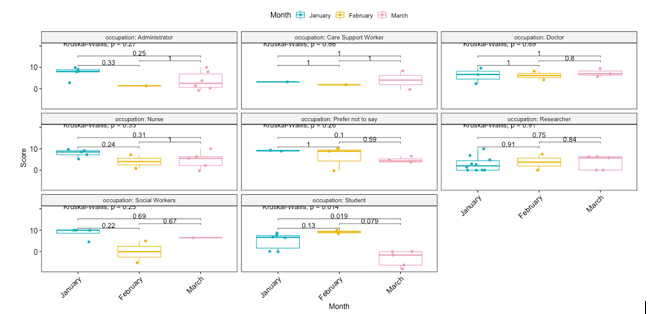


Supplementary Figure 10. Change in scores for the Symptoms questions by “occupation”.


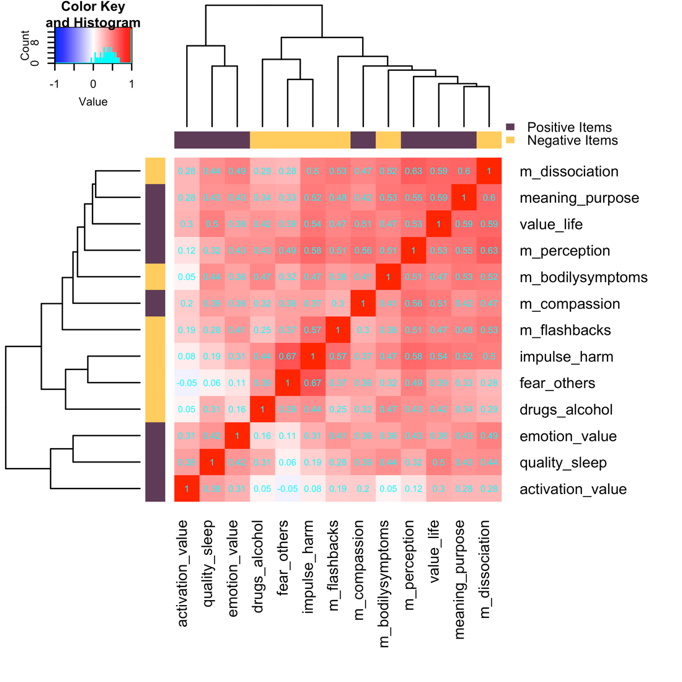

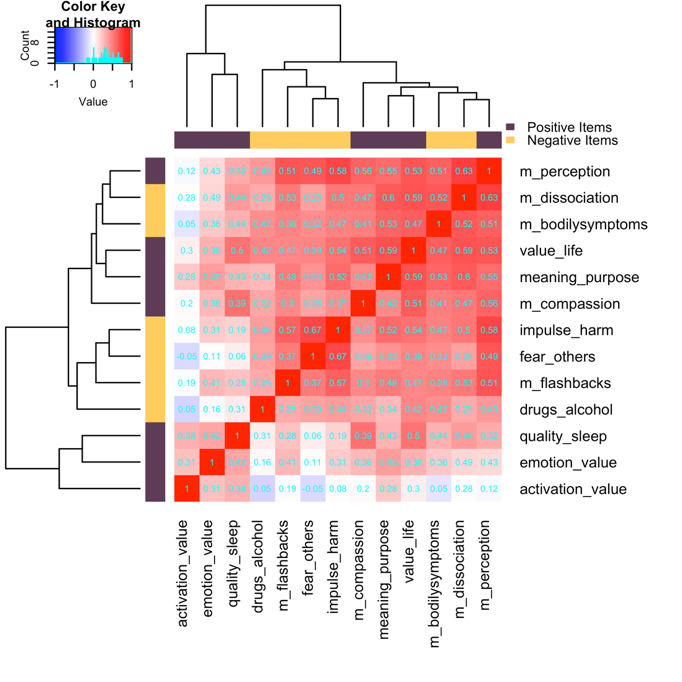


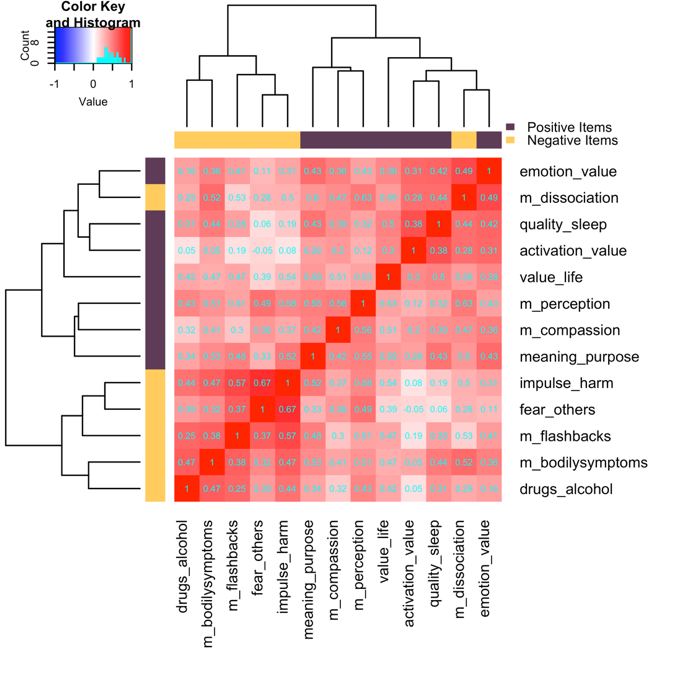

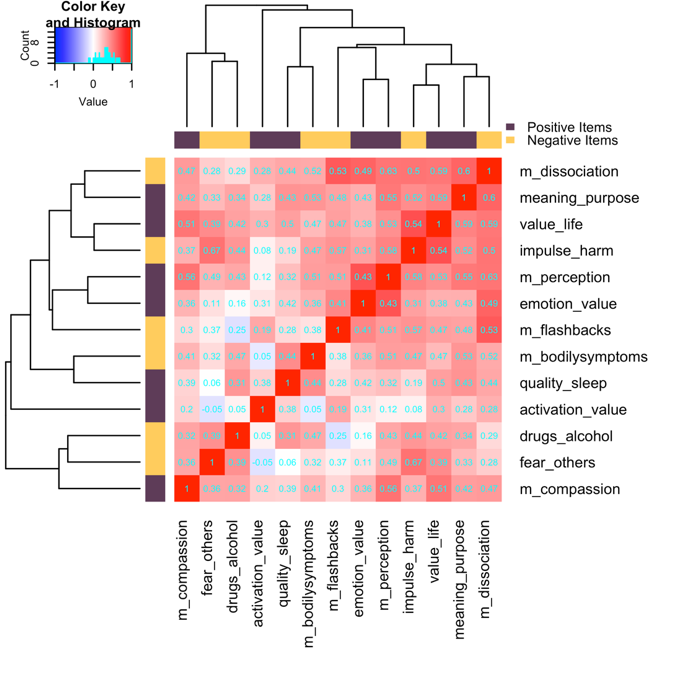


Supplementary Figure 11. Correlation scores for (A) All months are combined, (B) for January only, (C) for February only, (D) for March only.
